# Supplementary material for: Discovery and Functional Characterization of Two Regulatory Variants Underlying Lupus Susceptibility at 2p13.1
Source: Genes (Basel). 2022 Jun 5;13(6):1016. doi: 10.3390/genes13061016 (PMC9222795; doi:10.3390/genes13061016)
Supplement: Supplementary file 1 [file genes-13-01016-s001.zip › genes-1708315-supplementary/Figure S1-2.pdf]

Discovery and functional characterization of two regulatory variants underlying lupus susceptibility at 2p13.1

A

GM18502	DGUOK-AS1 expression							
DGUOK expression	0	1	2	3	4	5	6	9
1	3	2	1	0	0	-1	-1	-1
2	3	2	1	0	0	-1	-1	-1
3	3	2	1	1	0	-1	0	-1
4	3	2	1	1	0	-1	-1	-1
5	3	2	1	1	-1	-1	-1	0
6	2	2	1	1	-1	0	0	-1
7	2	2	1	1	-1	-1	-1	-1
8	2	2	1	1	-1	-1	-1	-1
9	2	1	1	1	-1	-1	-1	-1
10	2	1	1	0	-1	-1	-1	-1
11	1	1	1	0	-1	-1	-1	-1
12	1	1	0	-1	-1	-1	-1	-1
13	1	0	0	0	0	-1	-1	-1
14	0	0	-1	0	-1	-1	-1	-1
15	-1	-1	-1	0	-1	-1	-1	-1

B

GM12878	DGUOK-AS1 expression					
DGUOK expression	0	1	2	3	4	5
1	3	2	0	-1	-1	-1
2	3	2	1	0	-1	-1
3	3	2	1	0	-1	-1
4	3	2	1	-1	-1	-1
5	3	2	1	0	-1	-1
6	2	2	1	0	0	0
7	2	2	1	0	-1	-1
8	2	1	0	-1	-1	-1
9	2	1	0	-1	-1	-1
10	1	1	-1	-1	-1	-1
11	1	1	-1	-1	-1	-1
12	1	1	-1	-1	-1	-1
13	1	0	-1	-1	-1	-1
14	0	0	-1	-1	-1	-1
15	0	0	-1	-1	-1	-1

C

LCL777B958	DGUOK-AS1 expression						
DGUOK expression	0	1	2	3	4	5	6
1	3	1	-1	-1	-1	-1	-1
2	3	1	1	-1	-1	-1	0
3	2	1	0	-1	-1	-1	-1
4	2	1	0	0	-1	-1	-1
5	2	1	1	0	-1	-1	-1
6	2	1	0	-1	-1	-1	-1
7	2	1	0	-1	-1	-1	-1
8	1	1	0	0	-1	0	-1
9	1	0	-1	-1	-1	-1	-1
10	1	1	-1	-1	-1	-1	-1
11	1	1	-1	0	-1	-1	-1
12	1	1	-1	-1	-1	-1	-1
13	0	-1	-1	-1	-1	-1	-1
14	0	-1	-1	-1	-1	-1	-1
15	-1	-1	-1	-1	-1	-1	-1

D

LCL777B958-M81	DGUOK-AS1 expression						
DGUOK expression	0	1	2	3	4	5	6
1	3	2	0	-1	-1	-1	-1
2	3	2	1	-1	-1	-1	0
3	3	2	1	0	-1	-1	-1
4	3	2	0	-1	-1	-1	-1
5	2	1	1	0	-1	-1	-1
6	2	1	1	-1	-1	-1	-1
7	2	1	0	-1	-1	-1	-1
8	1	1	0	-1	-1	0	-1
9	1	1	0	-1	-1	-1	-1
10	1	1	0	-1	-1	-1	-1
11	1	1	-1	0	-1	-1	-1
12	0	0	-1	-1	-1	-1	-1
13	1	-1	-1	-1	-1	-1	-1
14	0	-1	-1	-1	-1	-1	-1
15	0	-1	-1	-1	-1	-1	-1

Supplementary Figure S1. Relationship between expression levels of *DGUOK* and *DGUOK-AS1* across four Coriell lines studied with single-cell RNA-seq

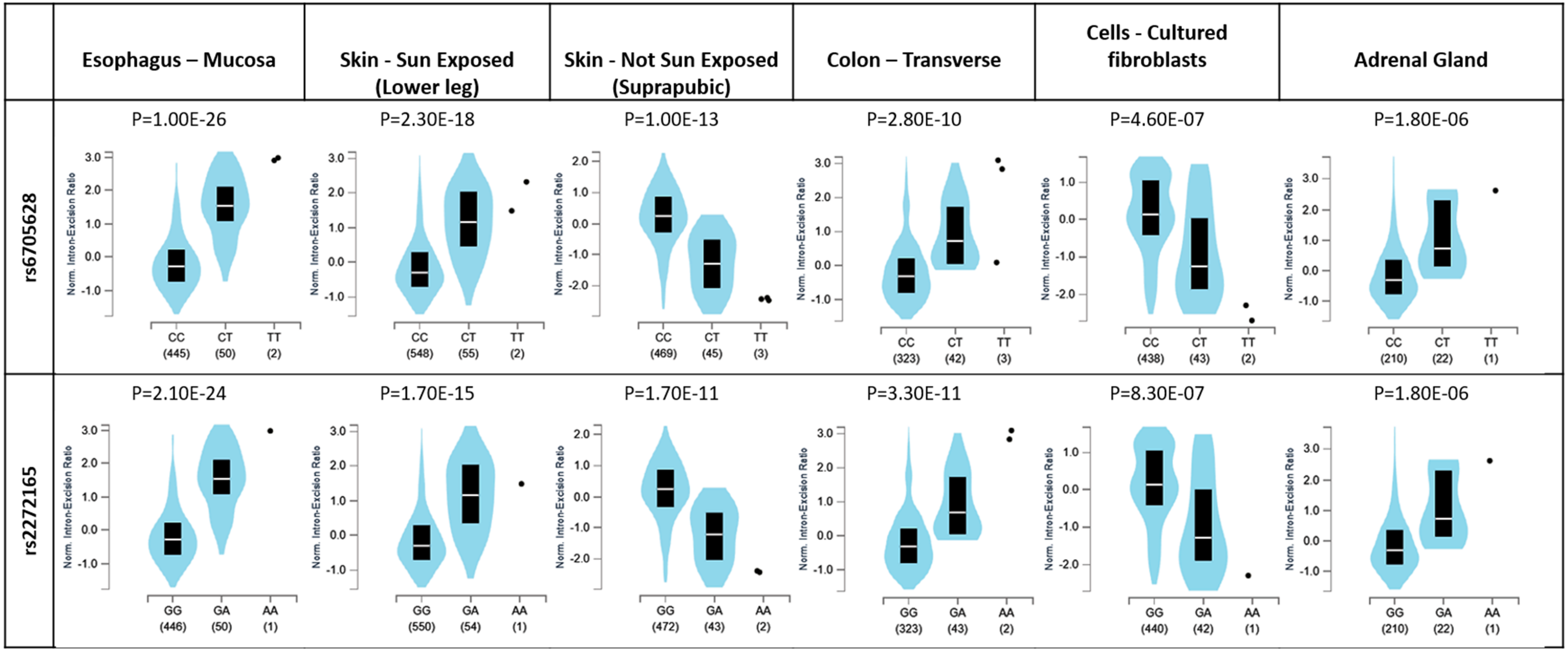

Supplementary Figure S2. GTeX (V8) data show significant sQTLs to anti-sense RNA *DGUOK-AS1* across multiple tissues with rs6705628 (top row) and rs2272165 (bottom row)
